# Daily electronic monitoring of subjective and objective measures of illness activity in bipolar disorder using smartphones– the MONARCA II trial protocol: a randomized controlled single-blind parallel-group trial

**DOI:** 10.1186/s12888-014-0309-5

**Published:** 2014-11-25

**Authors:** Maria Faurholt-Jepsen, Maj Vinberg, Mads Frost, Ellen Margrethe Christensen, Jakob Bardram, Lars Vedel Kessing

**Affiliations:** Psychiatric Center Copenhagen, Rigshospitalet, Blegdamsvej 9, DK-2100 Copenhagen, Denmark; The Pervasive Interaction Laboratory (PIT Lab), IT University of Copenhagen, Copenhagen, Denmark; Monsenso ApS, Rued Langgaards Vej 7, 2300 Copenhagen, Denmark

**Keywords:** Bipolar disorder, Randomized controlled trial, Smartphone, Depressive and manic symptoms, Illness activity, The MONARCA II trial, The Monsenso system, Electronic monitoring

## Abstract

**Background:**

Patients with bipolar disorder often show decreased adherence with mood stabilizers and frequently interventions on prodromal depressive and manic symptoms are delayed.

Recently, the MONARCA I randomized controlled trial investigated the effect of electronic self-monitoring using smartphones on depressive and manic symptoms. The findings suggested that patients using the MONARCA system had more sustained depressive symptoms than patients using a smartphone for normal communicative purposes, but had fewer manic symptoms during the trial. It is likely that the ability of these self-monitored measures to detect prodromal symptoms of depression and mania may be insufficient compared to automatically generated objective data on measures of illness activity such as phone usage, social activity, physical activity, and mobility. The Monsenso system, for smartphones integrating subjective and objective measures of illness activity was developed and will be tested in the present trial.

**Methods:**

The MONARCA II trial uses a randomized controlled single-blind parallel-group design. Patients with bipolar disorder according to ICD-10 who previously have been treated at the Copenhagen Clinic for Affective Disorder, Denmark are included and randomized to either daily use of the Monsenso system including an feedback loop between patients and clinicians (the intervention group) or to the use of a smartphone for normal communicative purposes (the control group) for a 9-month trial period. The trial was started in September 2014 and recruitment is ongoing. The outcomes are: differences in depressive and manic symptoms; rate of depressive and manic episodes (primary); automatically generated objective data on measures of illness activity; number of days hospitalized; psychosocial functioning (secondary); perceived stress; quality of life; self-rated depressive symptoms; self-rated manic symptoms; recovery; empowerment and adherence to medication (tertiary) between the intervention group and the control group during the trial. Ethical permission has been obtained. Positive, neutral and negative findings will be published.

**Discussion:**

If the system is effective in reducing depressive and/or manic symptoms (and other symptoms of bipolar disorder) and the rate of episodes, there will be basis for extending the use to the treatment of bipolar disorder in general and in larger scale.

**Trial registration:**

ClinicalTrials.gov NCT02221336. Registered 26^th^ of September 2014.

## Background

Patients with bipolar disorder often show decreased adherence with mood stabilizers [1–3] and frequently intervention on prodromal depressive and manic symptoms is delayed [4,5]. During recent years there have been an increasing growth of e-mental health technologies [6], and the amount of electronic platforms for self-monitoring in mental health, including bipolar disorder, is increasing rapidly. Recently, electronic self-monitoring of depressive and manic symptoms using regular cell phones to prompt patients with bipolar disorder to respond to weekly text messages has been suggested as an easy and inexpensive way to continuously monitor and identify prodromal depressive and manic symptoms and in this way allowing for health care providers to intervene shortly after symptoms first appear [7].

Other articles reporting on electronic self-monitoring have used personal digital assistants (PDAs) [8,9], computers [10–18] and smartphones [19] as the electronic self-monitoring tools, but none of the studies have however included data on objective measures of illness activity and the effect of electronic self-monitoring has only been investigated sparingly in randomized controlled trials (RCT) [20]. It is likely that the ability of these electronically self-monitored subjective measures may not be sufficient to detect prodromal depressive and manic symptoms compared to automatically generated behavioural data on measures of illness activity (objective measures) such as phone usage, social activity, physical activity, and mobility. Social activity [21], i.e., engaging in relations to others, as well as physical activity [22–24] represent central and sensitive aspects of illness activity in bipolar disorder, but none of the articles used patients monitored during non-experimental daily life, in naturalistic settings and with collection of real-time data.

Our group developed and tested the MONitoring, treAtment and pRediCtion of bipolAr disorder episodes system (the MONARCA system), an Android smartphone-based electronic self-monitoring system, in a number of studies during recent years [25–28]. The MONARCA system allowed for electronic subjective self-monitoring of mood, sleep, activity level, irritability, stress, medicine intake, alcohol consumption and other subjective personal measures and included a bi-directional feedback loop between patients and health care providers. The system also collected automatically generated behavioural data on measures of illness activity, such as accelerometer data, the number of incoming and outgoing phone calls/ day, and the number of incoming and outgoing text messages/ day [27].

The initial MONARCA pilot studies showed a high acceptance of the system and a higher compliance to self-monitoring than when monitoring on a paper-based version [25–28]. Further studies showed that electronic self-monitoring of depressive and manic symptoms using the MONARCA system correlated with observer-based clinically rated depressive and manic symptoms using the Hamilton Depression Rating Scale 17 item (HDRS-17) [29] and the Young Mania Rating Scale (YMRS) [30], respectively. Furthermore, HDRS-17 and YMRS correlated with a number of automatically generated behavioural data on measures of illness activity collected by the smartphone (e.g. the number and duration of incoming and outgoing calls/day and the number of outgoing text-messages/day) [28,31].

The MONARCA I RCT investigated the effect of daily electronic self-monitoring of subjective measures using the MONARCA system including a bi-directional feedback loop between the patient and clinicians compared with using a smartphone for normal communicative purposes in patients with bipolar disorder [20]. Overall no differences between the intervention group and the control group was found, but findings from sub-analyses suggested that patients using the MONARCA system had more sustained depressive symptoms than the control group and fewer manic symptoms in periods with presence of manic symptoms [20].

It has never been tested in a RCT whether electronic self-monitoring of subjective measures including a feedback loop integrating subjective as well as automatically generated behavioural data on measures of illness activity in patients with bipolar disorder improves illness outcome.

### Hypotheses

Using a smartphone-based monitoring system for daily electronic self-monitoring including an integrated feedback loop on both subjective and automatically generated behavioural data on measures of illness activity (phone usage, social activity, physical activity, and mobility) (the Monsenso system) reduces the severity of depressive and manic symptoms and rate of depressive and manic episodes in adult patients with bipolar disorder more than standard treatment.

### Objectives

To investigate in a randomized controlled single-blind parallel-group trial whether the use of a smartphone-based monitoring system including an integrated feedback loop on both subjective and automatically generated behavioural data on measures of illness activity (phone usage, social activity, physical activity, and mobility), the Monsenso system, reduces depressive and manic symptoms, the rate of depressive and manic episodes, the total number of days hospitalized, and improves psychosocial function, perceived stress, quality of life, self-rated depressive symptoms, self-rated manic symptoms, recovery, empowerment, and medicine adherence more than standard treatment in adult patients with bipolar disorder.

## Methods

The trial protocol is reported according to the CONsolidated Standards Of Reporting Trials (CONSORT) statement and Standard Protocol Items: Recommendations for Interventional Trials (SPIRIT) [32–34].

The trial protocol describes a randomized controlled single-blind parallel-group trial investigating the effect of using the Monsenso system on a daily basis, this including an integrated feedback loop, compared with using a smartphone for normal communicative purposes in adult patients with bipolar disorder.

### Trial design and study organization

**Figure 1:**
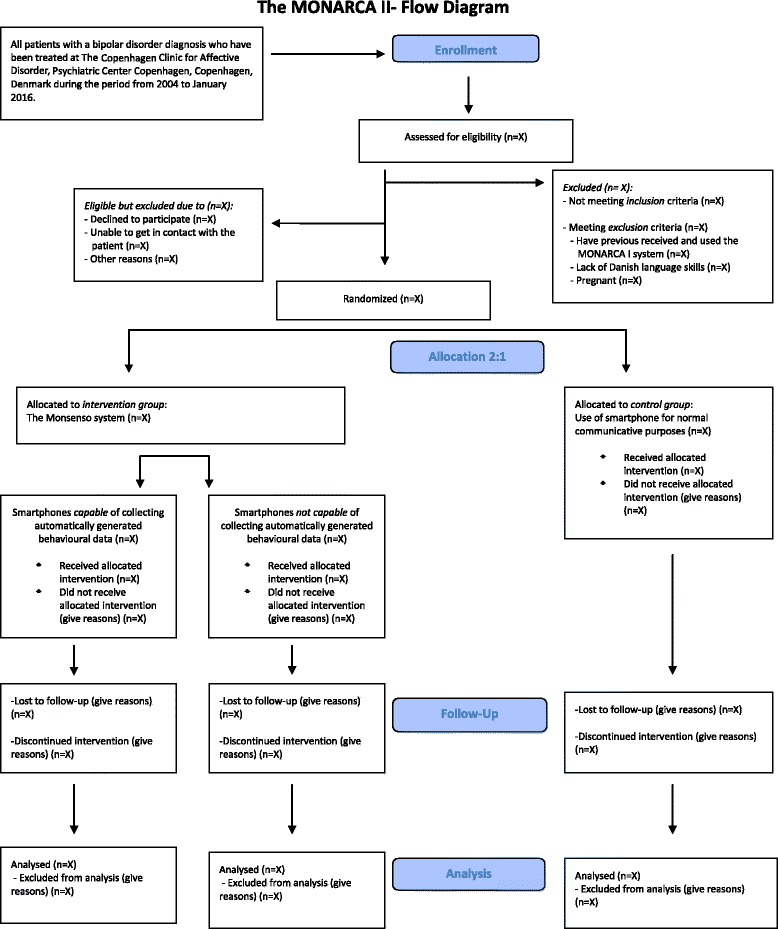
**The MONARCA II- Flow Diagram.**

The MONARCA II trial is a randomized controlled single-blind parallel-group trial with an unbalanced allocation ratio (2:1) of adult patients with bipolar disorder. The included patients are randomized to either active use of the Monsenso system on either an smartphone capable of collecting automatically generated behavioural data on measures of illness activity (e.g. Android smartphones) or an smartphone not capable of collecting automatically generated behavioural data on measures of illness activity (e.g. iPhone) (which type of smartphone used is chosen by the patients themselves, and is estimated to be approximately 50% on each of these types of smartphones) (the intervention group) or to the use of a smartphone for normal communicative purposes (the control group). The flow diagram of the MONARCA II trial is presented in Figure 1.

The study is conducted at the Psychiatric Center Copenhagen, Rigshospitalet, Copenhagen, Denmark. No changes in study design or methods have been made after trial commencement.

### Participants and settings

All patients with a diagnosis of bipolar disorder according to ICD-10 who have been treated and discharged from The Copenhagen Clinic for Affective Disorder, Denmark in the period 2004 to January 2016 are invited to participate in the trial. This corresponds to approximately 400 potential participants. The Copenhagen Clinic for Affective Disorder is a specialized outpatient clinic that covers a recruitment area of the Capital Region, Denmark, corresponding to 1.4 million people. The staff consists of full-time specialists in psychiatry with a specific clinical experience and knowledge about the diagnosis and treatment of bipolar disorder as well as certified psychologist, nurses and a social worker with experience in bipolar disorder. Patients with bipolar disorder were referred to the Copenhagen Clinic for Affective Disorder from secondary healthcare when a diagnosis of a single mania or bipolar disorder was made for the first time or in the case of occurrence of treatment resistance (persistent depressive or manic symptoms or recurrence despite treatment in standard care). The physicians at the clinic followed the patients with evidence-based psychopharmacological treatment and regular appointments. Treatment at the Copenhagen Clinic for Affective Disorder comprised combined psychopharmacological treatment as according to the guidelines from the British Association for Psychopharmacology [35] and supporting therapy for a 2-year period [36].

Inclusion criteria: All patients between the age of 18 to 75 years with a bipolar disorder diagnosis according to ICD-10 using Schedules for Clinical assessments in Neuropsychiatry (SCAN) [37] who have been treated and discharged from The Copenhagen Clinic for Affective Disorder, Denmark from the period 2004 to January 2016 are invited to participate in the MONARCA II trial.

Exclusion criteria: Patients who have received the MONARCA system as part of previous studies are excluded. Patients who are pregnant and with a lack of Danish language skills are excluded.

Patients meeting the inclusion criteria and having none of the exclusion criteria are invited to be enrolled in the MONARCA II trial.

### Study procedure

Potential participants are invited to participate in the MONARCA II trial first by mail, then by phone calls and lastly by e-mail if no responses are received via the first two methods. All potential participants who accept to meet with the researcher (MFJ) for further trial information are screened if they fulfill the criteria for participation and then included in the MONARCA II trial. Following inclusion, baseline assessments are performed on all patients and after these assessments are the numbered opaque allocation envelopes distributed by a research secretary (HGN) to the MONARCA II study nurse and the patients are randomized to either the intervention group or the control group for a 9-month trial period. An overview of the assessments is presented in Table 1.

**Table 1:** **Outcome assessment overview- The MONARCA II trial**

	**SCAN** ^**a**^	**Baseline characteristics**	**Rating scales** ^**b**^	**Questionnaires** ^**c**^	**Clinical information** ^**d**^	**Biological samples** ^**e**^
Inclusion and baseline	x	x	X	X	x	X
Randomization (2:1) to using a smartphone with the MONARCA II system (the intervention group) or to use a smartphone for normal communicative purposes (the control group)						
4 weeks			X	X	x	X
3 months			X	X	x	X
6 months			X	X	x	x
9 months			X	X	x	x

^a^Schedules for Clinical Assessment in Neuropsychiatry interview (37).

^b^Depressive symptoms according to Hamilton Depression Rating Scale 17-item (29), manic symptoms according to Young Mania Rating Scale (30) and psychosocial functioning according to Functioning Assessment Short Test (FAST) (38).

^c^Perceived stress according to Cohen’s Perceived Stress scale (39), quality of life according to WHO Quality of Life-BREF (WHOQOL-BREF) (40), self-rated depressive symptoms according to Becks Depressive Inventory (BDI) (41–43), self-rated manic symptoms according to Altman Self Rating scale for Mania (ASRM) (44), recovery according to Recovery Assessment Scale (45), empowerment according to Rogers empowerment scale (47) and medicine adherence according to Medicine Adherence Rating Scale (MARS) (46).

^d^Number of affective episodes, number of contacts to clinicians and psychiatric emergency rooms, number of hospitalizations, medication status etc.

^e^Blood samples is obtained by venipuncture, 90 ml in total and a freshly voided spot urine sample is collected.

### Interventions

All included patients have received treatment and have been discharged from The Copenhagen Clinic for Affective Disorder, Denmark during the period from 2004 to January 2016 at inclusion in the present MONARCA II trial. All included patients continue their treatment as usual at a community psychiatric centre, a private psychiatrist, a general practitioner or outpatient treatment at a hospital during the trial period.

#### The MONARCA studies

Following the MONARCA studies adjustments to the self-monitoring part of the system were made and a new integrated feedback loop, based on prediction models including both subjective measures and automatically generated behavioural data on measures of illness activity, was established. This is the Monsenso system, and the effect of this is investigated in the present MONARCA II trial.

#### The smartphones

In the MONARCA II trial the Monsenso system is available for smartphones capable of collecting automatically generated behavioural data on measures of illness activity (e.g. different versions of Android smartphones) or smartphones not capable of collecting automatically generated behavioural data on measures of illness activity (e.g. iPhones). All patients are, regardless of randomization allocation, offered to loan an Android smartphone free of charge for the 9-month trial period. The patients, regardless of randomization allocation, who are using a smartphone not capable of collecting automatically generated behavioural data on measures of illness activity beforehand and do not want to loan and use the Android smartphones offered by the MONARCA II trial, are offered to use their own smartphones for the 9-month trial period.

The patients in the intervention group have to use the Monsenso system for daily electronic self-monitoring, and the patients in the control group have to use the smartphones for normal communicative purposes. This is regardless the choice of smartphone type. Both the intervention group and the control group have to use the smartphones for the 9-month trial period. Economic costs from data traffic due to the MONARCA II trial are refunded to all participants regardless of randomization allocation and choice of smartphone type.

#### Subjective (self-monitored) measures of illness activity in the intervention group

The patients randomized to the intervention group, regardless the choice of smartphone, are prompted by an alarm in the Monsenso system at a self-chosen time during the day to evaluate subjective measures of illness activity on a daily basis. The following subjective (self-monitored) measures of illness activity are available for daily evaluation: mood (scored from depressive to manic on a scale from −3, −2, −1, −0.5, 0, +0.5, +1, +2, +3), sleep duration (number of hours slept per night, measured in half-hour intervals), medicine intake (taken as prescribed/ taken with changes (if changes, the patients are asked to specify these)/not taken), activity level (scored from very low to very high on a scale from −3, −2, −1, 0, +1, +2, +3), mixed mood (yes/no), irritability (scored from not present, present to some degree or present on a scale from 0, 1, 2), anxiety (scored from not present, present to some degree or present on a scale from 0, 1, 2), cognitive problems (scored from not present, present to some degree or present on a scale from 0, 1, 2), alcohol consumption (number of units consumed per day, 0 to +10 scale), stress (scored from not present, present to some degree or present on a scale from 0, 1, 2), menstruation for women (yes/no), individual early warning signs (yes/no), a number (unlimited) of personal parameters (created by the patients themselves), and a free-text note.

**Figure 2:**
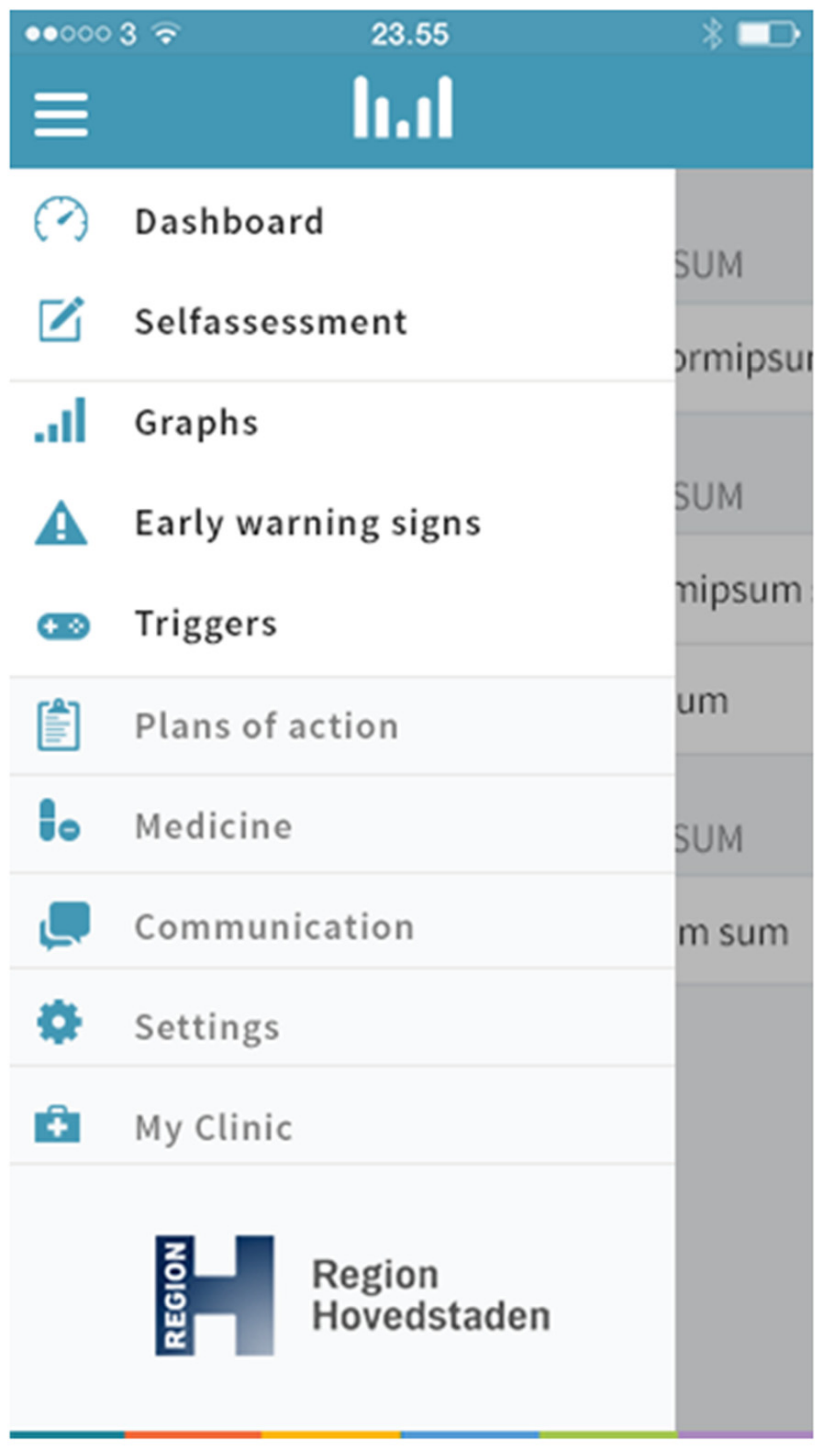
**The Monsenso system, dashboard.**

**Figure 3:**
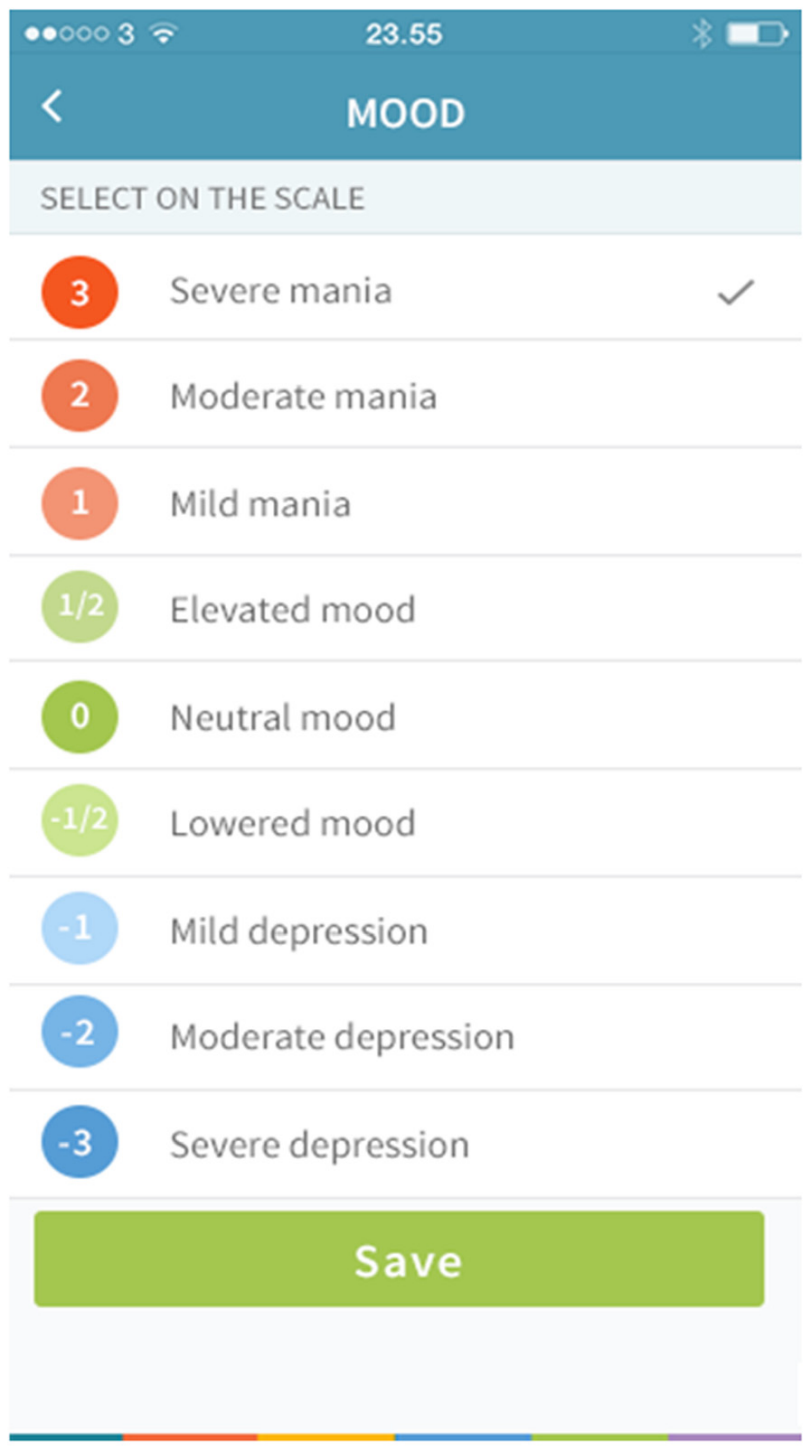
**The Monsenso system, self-monitored mood.**

After midnight, the entered subjective (self-monitored) measures of illness activity are “locked” and further changes cannot be made. If the patients wish to change their subjective evaluation they can enter a second evaluation in addition to the initial one, and both of the subjective evaluations are then visible for the patient and the health care provider when logging on to in the Monsenso system. If the patients forget to evaluate the subjective measures it is possible to enter and evaluate retrospectively for up to two days. It is then noted in the Monsenso system that the subjective measures are collected retrospectively. Screenshots from the Monsenso software are presented in Figures 2 and 3. A user’s guide for the Monsenso system was developed and is handed out to all patients in the intervention group (can be obtained by contacting the first author).

#### Automatically generated behavioural data (objective data) on measures of illness activity in both the intervention group and the control group

All of the smartphones capable of collecting automatically generated behavioural data on measures of illness activity regardless of randomization group (intervention group or control group) collects as many automatically generated behavioural data on measures of illness activity (objective data) on a daily basis during the 9-month trial period as possible (different smartphones hardware and operating systems support different types of sensors) (Figure 1).

Examples of some of the automatically generated behavioural data on measures of illness activity collected by the smartphones: 1) phone usage measured as the amount of time the smartphones screen are turned on/off, battery usage, ambient light, and ‘proximity’ detection 2) social activity measured as the number of in- and outgoing phone calls and text messages, the duration of in- and outgoing phone calls, the length of the text messages, and the time of the day when the phone calls and/or text messages are made/send/or received 3) physical activity measured by the step counter in the smartphones, and 4) mobility based on the location estimation available in the smartphones (which again rely on e.g. GPD or GSM cell tower information depending on specific circumstances). Furthermore, speech activity is collected by extraction of different voice features during phone calls. Voice feature extraction will take place directly on the smartphones and no recording of the actual speech/conversation will take place.

Thus, we will be able to investigate correlations between automatically generated behavioural data on measures of illness activity and the severity of depressive and manic symptoms of all participants in the trial, using smartphones capable of collecting automatically generated data on measures of illness activity, including the ones allocated by randomization to the control group.

#### The integrated feedback loop between patients and clinicians in the intervention group

A study nurse with experience with bipolar disorder is assigned to the patients allocated to the intervention group of the MONARCA II trial. The MONARCA II study nurse is responsible for the integrated feedback loop. Patients allocated to the intervention group of the MONARCA II trial have the Monsenso application installed on a smartphone, and this automatically transfers the self-monitored subjective measures and for some smartphones also the automatically generated behavioural data on measures of illness activity (Flow diagram in Figure 1) to servers at the hospital through secure connections (I-suite number RHP-2011-03). By giving informed consent to participate in the MONARCA II trial, the patients allow for the MONARCA II study nurse and their health care provider to access the monitored data through a secure web interface. The MONARCA II study nurse goes through the collected data two to three times a week, or more often on patients where it is deemed necessary. A personal homepage is set up on a server allowing for the patients to access all their own data through a similar secure web interface.

1. The feedback loop on subjective measures: Regardless the choice of smartphone a feedback loop on the subjective measures is established. A standard of scoring thresholds for when the MONARCA II study nurse initially should react was made. For example, the MONARCA II study nurse reacts if the patients register ≥ −2 on the mood item for two days or more, or if the patients register changes in their sleep patterns of 1 hour or more for more than three days. Following a run in phase of approximately two to four weeks of self-monitoring, the patients and the MONARCA II study nurse individualize the thresholds for when reaction should be made. Also the MONARCA II study nurse and the patients agree on a concordance status in a) the patients most important items for identifying prodromal symptoms of depression as well as (hypo)mania b) the threshold for future early warning signs c) actions to be taken in case of depression or (hypo)mania.
2. The integrated feedback loop on subjective and automatically generated behavioural data on measures of illness activity: A feedback loop integrating subjective and automatically generated behavioural data on measures of illness activity is established for patients using smartphones capable of collecting automatically generated data on measures of illness activity (Figure 1). The feedback loop integrates both subjective and automatically generated behavioural data on measures of illness activity in a flexible and adjustable model (a learning system) resulting in prediction analyses of the collected data providing messages for both the patients and the MONARCA II study nurse such as: “you should contact the MONARCA II study nurse”.

Actions by the study nurse as part of the feedback loop in the intervention group: In the case of signs of deterioration of a patient the MONARCA II study nurse a) contacts the patient and give advice on how to handle the situation b) (if the first action is not enough) asks the patient to contact his/her usual physician or other clinician c) (if the above actions are not enough, or if contact to the patient is not possible) contacts the patient’s usual physician or other clinician d) (if acute deterioration and/ or severe symptoms) contacts the psychiatric emergency service in Copenhagen, Denmark.

### Assessments

All assessments are carried out by one researcher (MFJ) who is not involved in the treatment of the patients. The bipolar disorder diagnoses according to ICD-10 are confirmed by a SCAN interview before inclusion of the patients. The patients are, regardless of randomization group, enrolled for a 9-month trial period and invited for outcome assessments by a blinded researcher (MFJ) at baseline, after 4 weeks, after 3 months, after 6 months and after 9 months (Table 1).

At each visit with the researcher the assessments include the following: The severity of depressive and manic symptoms is measured using HDRS-17 item and YMRS, respectively, and psychosocial functioning is measured using the Functioning Assessment Short Test (FAST) [38]. The following questionnaires are fulfilled when visiting the researcher: perceived stress according to Cohen’s Perceived Stress scale [39], quality of life according to WHO Quality of Life-BREF (WHOQoL-BREF) [40], self-rated depressive symptoms according to Becks Depressive Inventory (BDI) [41–43], self-rated manic symptoms according to Altman Self Rating scale for Mania (ASRM) [44], recovery according to Recovery Assessment Scale (RAS) [45], medicine adherence according to Medicine Adherence Rating Scale (MARS) [46] and empowerment according to Rogers Empowerment Scale (RES) [47].

Furthermore, the patients are asked to show up fasting between 8–10 A.M. at baseline, after 4 weeks, 3 months, 6 months and 9 months when visiting the researcher and the following biological samples are collected: 1) Blood samples obtained by venipuncture, 90 ml in total and 2) a freshly voided morning spot urine sample (around 20 ml). The biological samples will be analyzed at the end of the trial for candidate biological markers that are potentially related to alterations in illness activity and affective state in bipolar disorder.

### Outcomes

#### Primary outcomes

- Differences in clinically rated depressive and manic symptoms measured using HDRS-17 and YMRS, respectively, between the intervention group and the control group.
- Differences in depressive and manic symptoms measured using HDRS-17 and YMRS, respectively, between the intervention group and the control group in patients with presence of depressive and manic symptoms (defined as HDRS-17 > 0 or YMRS > 0) *at any given visit* with the researcher during the 9-month trial period.
- Differences in depressive and manic episodes defined as HDRS-17 ≥14 or YMRS ≥14 between the intervention group and the control group.

#### Secondary outcomes

- Differences in automatically generated behavioural data (objective data) collected from the smartphones (e.g. mobility, social and physical activity etc.) between the intervention group and the control group.
- Differences in the total number of days hospitalized between the intervention group and the control group.
- Differences in psychosocial functioning according to the FAST score between the intervention group and the control group.

#### Tertiary outcomes

Differences in perceived stress according to Cohen’s Perceived Stress scale, quality of life according to the WHOQOL-BREF score, self-rated depressive symptoms according to BDI, self-rated manic symptoms according to ASRM, recovery according to the Recovery Assessment Scale, empowerment according to Rogers Empowerment Scale and medicine adherence according to MARS between the intervention group and the control group.

No changes in trial outcomes have been made after trial commencement.

### Statistical power and sample size calculation

The statistical power and sample size was calculated using http://stat.ubc.ca/~rollin/stats/ssize/n2.html.

The primary outcomes are a) differences in the level of depressive and manic symptoms based on HDRS-17 and YMRS, respectively and b) differences in the number of depressive and manic episodes defined as HDRS ≥14 and YMRS ≥14, respectively.

- The clinical relevant difference in the level of depressive and manic symptoms is defined as a minimum of three scores on HDRS-17 and YMRS, respectively, and the standard deviation (SD) is set to 7 with a mean score of 7 vs. 10 in the intervention group and the control group, respectively. The statistical power to detect a three score difference in the areas under the curves between the intervention group and the control group on HDRS-17 and YMRS, respectively, is 80% with α = 0.05 for a two-sample comparison of means including a minimum of 86 patients in each of the two sub-groups in the intervention group (smartphones capable of collecting automatically generated behavioural data or not) (n = 172) and 86 patients in the control group (n = 86). This results in an estimated total sample size of 258 patients.
- The clinical relevant difference in the number of depressive and manic episodes is defined as a minimum of 10% difference and the SD is set to 23% with a mean score of 40% vs. 50% in the intervention group and the control group, respectively. The statistical power to detect a 10% difference in the area under the curves between the intervention group and the control group on the number of depressive and manic episodes is 80% with α = 0.05 for a two-sample comparison of means including about the same number of patients in each of the two sub-groups in the intervention group (smartphones capable of collecting automatically generated behavioural data or not) (n = 172) and in the control group (n = 86).

### Randomization

#### Sequence generation

A computer-generated list of random allocation numbers using randomization.com was carried out by an independent researcher (KM), who is not a part of the trial. Patients included in the trial are randomized with an unbalanced allocation ratio of 2:1 to using the Monsenso system on a daily basis including the integrated feedback loop (based on either a) subjective self-monitored measures alone or 2) a combination of subjective self-monitored measures and automatically generated behavioural data on measures of illness activity) (the intervention group) or to the use of a smartphone for normal communicative purposes (the control group) (Figure 1). A design with an unbalanced allocation ration was chosen since the integrated feedback loop using smartphones capable of collecting automatically generated behavioural data on measures of illness activity or not is not based on the same amount of collected data, and thus comprising two versions of the integrated feedback loop. The choice of smartphone used is chosen by the patients themselves at inclusion *before* the randomization, and is estimated to by approximately 50% on each of these types of smartphones (smartphones capable of collecting automatically generated behavioural data on measures of illness activity (e.g. different versions of Android smartphones) or not (e.g. iPhones)). Thus, a design with an unbalanced allocation ration of 2:1 will result in three groups of approximately 86 patients in each group (Figure 1).

Since the MONARCA II trial is single-blinded, random block sizes are used to help preserve unpredictability [48,49]. The MONARCA II study nurse is unaware of the range of numbers of patients in the random block sizes.

Since then gain from stratification becomes minimal when the number of participants in each group of a trial is more than 50 [50], but adds complexity, a non-stratified randomization design was chosen. The statistical analyses will however be adjusted for age and sex as possible prognostic variables.

#### Allocation concealment and implementation

The allocation sequence is concealed from the researcher (MFJ) enrolling and assessing the patients and from the MONARCA II study nurse. Allocation is concealed in numbered, opaque and sealed envelopes and stored in a securely locked cabinet of unknown location to others than the research secretary (HGN). Allocation is identified by the letter A or B written on the paper (different colour of paper for the two different randomization groups) inside the envelopes and this indicate the allocation to intervention (intervention group or control group). The translation of the letters A and B was made and known to only KM, LVK and the MONARCA II study nurse. A paper with this translation is kept in a securely locked cabinet of unknown location to others than KM and LVK. The secretary (HGN) gives the allocation envelopes to the MONARCA II study nurse after enrolment and baseline assessments of the patients. Corresponding allocation envelopes are opened only after all baseline assessments are performed and the patients’ names and social security numbers are written on both the envelopes and the randomization papers. The MONARCA II study nurse assigns patients to their allocation of intervention.

### Blinding

Owing to the type of intervention in the MONARCA II trial, the patient, the patients’ health care provider and the MONARCA II study nurse are aware of the allocated randomization group. The researchers responsible for outcome assessments, data entry, data analysis, interpretation of analysis and writing of papers are kept blinded to allocation at all times during the trial period, data analysis and interpretation of analysis. The trial is therefore single-blinded. The MONARCA II study nurse does not collect any outcome measures. All patients are thoroughly and at each visit with the researcher instructed not to mention anything about randomization allocation. The risk of unblinding due to simply seeing the patients’ smartphone is minimized since all patients use a smartphone during the trial period.

### Statistical methods

Data from all randomized patients are collected until dropout or the end of the trial period. Analysis will be carried out with an intention-to-treat (ITT) approach. The primary outcomes are differences in depressive and manic symptoms using HDRS-17 and YMRS, respectively, during the 9-month trial period. Analysis will be done employing a linear mixed effects model with random intercept for each participant and a fixed effect of visit number. Differences in outcomes between the intervention group and the control group will be analyzed, firstly in an unadjusted model (except for potential differences in baseline values of the outcome variable) and then in models adjusted for age and sex as possible prognostic variables.

Furthermore, analysis will be done employing a linear mixed effects model with random intercept for each participant and a fixed effect of visit on differences in HDRS-17 and YMRS in patients with presence of depressive and manic symptoms (defined as HDRS-17 > 0 or YMRS > 0) at a given time point during the trial period between the intervention group and the control group. Additionally, analysis of the primary outcomes of differences in depressive and manic episodes defined as HDRS ≥14 and YMRS ≥14 during the trial period will be done employing survival analysis with reasons for censoring being date of death or date of drop out.

Potential interactions between randomization group (intervention group or control group) and visit number in the analyses will be investigated and reported accordingly. The statistical threshold for significance is p ≤ 0.05 (two-tailed). Data will be managed by MFJ and entered using Epidata® (Epidata Association, Odense, Denmark). All analyses will be done using STATA version 12 (StataCorp LP, College Station, TX, USA).

### Ethical considerations and approval

Ethical permission for the MONARCA II trial has been obtained from the Regional Ethics Committee in The Capital Region of Denmark and The Danish Data Protection Agency (H-2-2014-059). The law on handling of personal data will be respected. The patients’ journals will only be read to confirm information regarding the patients’ clinical history. The trial was registered at ClinicalTrials.gov as NCT02221336 on the 26^th^ of September 2014. Link to the trial registration: https://clinicaltrials.gov/ct2/show/NCT02221336?term=MONARCA+II&rank=1. All positive, neutral and negative findings of the trial will be published according to the CONSORT guidelines [32]. All electronic monitored data are stored at a secure server at Capital Region, Copenhagen, Denmark. The usual health care provider/physician/clinician of the patients randomized to the intervention group are contacted by letter at the beginning of the trial and informed about the trial and that they might get contacted by the MONARCA II study nurse if there are signs of deterioration of the patient.

All potential participants are invited to receive information about the MONARCA II trial on an individual basis where the information is given in a quiet and undisturbed office. All information is presented in both written and verbal form and participants can bring a friend or relative to the introduction conversation. Participants are informed that participation is voluntary and that consent can be withdrawn at any time during the trial without this having any consequences for current and future treatment options. All participating patients sign a consent form and get a copy of this and their rights as a participant in a clinical trial. All participating patients are offered to loan a smartphone free of charge during the trial period, and economic costs due to data traffic from the Monsenso system are refunded. Participants do not receive any economic compensation for participating in the MONARCA II trial.

## Results

Recruitment is ongoing.

## Discussion

There are yet few studies on electronic self-monitoring in bipolar disorder, and the evidence of electronic self-monitoring has been sparingly investigated and is limited by methodological issues and by a lack of RCTs [51]. Although the idea of electronic self-monitoring seems appealing, studies using rigorous methodology investigating beneficial and possible harmful effects of electronic self-monitoring are needed.

Our group, consisting of psychiatrists, bipolar disorder researchers and IT experts, developed the MONARCA system in close collaboration with patients with bipolar disorder [25,26]. The MONARCA system included a feedback loop on subjective self-monitored measures between patients and clinicians and was tested by the authors in multiple studies, this including a RCT [20,27,28,31]. Subsequently a new version, the Monsenso system, was developed. The MONARCA II trial is the first trial investigating the effect of electronic self-monitoring of subjective measures including a feedback loop integrating subjective as well as automatically generated behavioural data on measures of illness activity on depressive and manic symptoms in patients with bipolar disorder.

### Limitations

#### The intervention

The MONARCA II trial is designed to investigate the effect of the total Monsenso system, comprising daily self-monitoring of subjective measures and an integrated feedback loop (based on either subjective self-monitored measures or a combination of subjective self-monitored measures and automatically generated data on measures of illness activity) between patients and health care providers, compared with using a smartphone for normal communicative purposes. Thus, it will not be possible to address the effect of the individual elements of the intervention.

The integrated feedback loop is based on prediction models based on self-monitored subjective self-monitored measures and for those smartphones capable of collecting these data also on automatically generated behavioural data on measures of illness activity. The integrated feedback loop and prediction models in the Monsenso system are therefore not based on the same amount of data in the two smartphone groups. The prediction models in the integrated feedback loop are adjusted during the trial period (a learning model), due to increasing technical possibilities, to achieve the best prediction possible.

#### The control group

As in any other non-pharmacological treatment trial is it always a challenge to define a proper control group. The MONARCA II trial is designed to include a control group of patients using a smartphone for normal communicative purposes, a placebo smartphone. Automatically generated behavioural data on measures of illness activity is collected on all patients using a smartphones capable of collecting these data during the trial period, including the patients in the control group, this allowing for correlation analyses between depressive and manic symptoms and the automatically generated behavioural data on measures of illness activity in a larger sample of patients with bipolar disorder.

### Generalizability

A RCT represents a study design with possible high interval validity, but this with a possible cost of lower external validity and thereby lower generalizability of the study results. The MONARCA II trial recruits patients who have received treatment at and been discharged from a tertiary specialized mood disorder clinic, however including patients with recent onset of bipolar disorder, whom in most countries are treated elsewhere. Further, the trial has a pragmatic design with few exclusion criteria. In addition, as the Monsenso system is user-friendly for both the patients and the health care providers, and previous MONARCA studies showed a high acceptance and compliance of the previous system [25–28], the findings of the MONARCA II trial are believed to be generalizable to patients with bipolar disorder in general.

### Perspectives

Electronic monitoring using smartphones represents a flexible and adjustable system that could be of great support for both patients and health care providers, and possibly increase the patients’ illness insight, empowerment and awareness of early warning signs of upcoming depressive or manic episodes.

If the Monsenso system is proved effective in reducing the level of depressive and/or manic symptoms (and other symptoms of bipolar disorder) and the rate of depressive and manic episodes in the present trial, there will be basis for extending the use of the system to the treatment of patients with bipolar disorder in general and in a larger scale. Potentially electronic monitoring using smartphones may be applied in relation to patients suffering from other mental disorders. In this way, it is possible that outpatient treatment in general can be optimized, and that the frequency of necessary health care provider/physician/clinician and other clinical staff visits can be reduced and be more flexible according to the needs of the patients.
